# The efficacy of virtual reality exposure therapy for the treatment of alcohol use disorder among adult males: a randomized controlled trial comparing with acceptance and commitment therapy and treatment as usual

**DOI:** 10.3389/fpsyt.2023.1215963

**Published:** 2023-08-22

**Authors:** Hongdu Deng, Ruiling Zhang, Chuansheng Wang, Bingyu Zhang, Jiali Wang, Shilin Wang, Jie Zhang, Nurul Izzah Shari, Mohammad Farris Iman Leong Bin Abdullah

**Affiliations:** ^1^Department of Community Health, Advanced Medical and Dental Institute, Universiti Sains Malaysia, Penang, Malaysia; ^2^Second Affiliated Hospital, Xinxiang Medical University, Henan, China; ^3^School of Human Resource Development and Psychology, Faculty of Social Sciences and Humanities, Universiti Teknologi Malaysia, Skudai, Johor, Malaysia

**Keywords:** alcohol use disorder, virtual reality exposure therapy, acceptance and commitment therapy, randomized controlled trial, craving

## Abstract

**Background:**

This multicenter, three-armed, parallel, single-blind randomized controlled trial (RCT) primarily aims to compare the efficacy of virtual reality exposure therapy (VRET) with that of acceptance and commitment therapy (ACT) and treatment as usual (TAU) to depreciate the degree of alcohol craving among alcohol use disorder patients who have undergone in-patient detoxification across four timelines (t_0_ = baseline prior to intervention, t_1_ = 4 weeks after baseline, t_2_ = 12 weeks after baseline, and t_3_ = 24 weeks after baseline). The secondary aims of this RCT are to compare the efficacy of VRET with that of ACT and TAU to alleviate the severity of alcohol use disorder, dissipate comorbid depressive and anxiety symptoms, and normalize event-related potential (ERP) in electroencephalogram (EEG) monitoring across the four timelines.

**Methods:**

Initially, after 2 weeks of in-patient detoxification, 120 patients with alcohol use disorder will be randomized into three groups (VRET, ACT, and TAU control groups) via stratified permuted block randomization in a 1:1:1 ratio. Baseline assessment (t_0_) commences, whereby all the participants will be administered with sociodemographic, clinical, and alcohol use characteristics questionnaire, such as Alcohol Use Disorder Identification Test (AUDIT), Penn Alcohol Craving Scale (PACS), Hamilton Anxiety Rating Scale (HAM-A), and Hamilton Depression Rating Scale (HAM-D), while event-related potential (ERP) detection in electroencephalogram (EEG) will also be carried out. Then, 4 weeks of VRET, ACT, and non-therapeutic supportive activities will be conducted in the three respective groups. For the subsequent three assessment timelines (t_1_, t_2_, and t_3_), the alcohol use characteristic questionnaire, such as AUDIT, PACS, HAM-D, HAM-A, and ERP monitoring, will be re-administered to all participants.

**Discussion:**

As data on the effects of non-pharmacological interventions, such as VRET and ACT, on the treatment of alcohol craving and preventing relapse in alcohol use disorder are lacking, this RCT fills the research gap by providing these important data to treating clinicians. If proven efficacious, the efficacy of VRET and ACT for the treatment of other substance use disorders should also be investigated in future.

**Clinical trial registration:**

NCT05841823 (ClinicalTrials.gov).

## 1. Introduction

Alcohol is an easily accessible substance that is associated with the risk of various morbidity and mortality. Alcohol consumption per capita in the world rose from 5.5 L in 2005 to 6.4 L in 2010, and it remained at 6.4 L in 2016. As alcohol consumption has not depreciated, in 2016, alcohol-related disability-adjusted life years (DALYs) stood at 132 million and mortality was at 3.0 million deaths worldwide. Hence, alcohol contributed to 5.0% of DALYs and 5.3% of all-cause mortality worldwide (1).

Similarly in China, alcohol use is on the rise, in which the prevalence of alcohol consumption among those aged 18 years and older rose from 35.7% in 2007 to 37.1% in 2013 and 41.3% in 2015, according to the China Chronic Disease and Nutrition Surveillance (CCDNS). Unfortunately, the alcohol-related burden of disease and injury increased rapidly in China from 1990 to 2017, in which the prevalence of DALYs and age-standardized DALY rate was at 58.51 and 16.02%, according to the Global Burden of Disease study. In 2013, China registered 381,200 alcohol-related mortality cases and alcohol caused reduced life expectancy of the Chinese people by 0.43 years (2).

In Malaysia, the National Health and Morbidity Survey (NHMS) 2011 revealed that the prevalence of current alcohol use was 11.6%, and among the alcohol users, 23.6% of them practiced hazardous drinking. Among the ethnicity, Malaysian Chinese is more prevalent to consume alcohol (3). The NHMS 2019 in Malaysia reported a similar prevalence of alcohol consumption in the country at 11.5%, and Malaysian Chinese remained as the most prevalent ethnicity to consume alcohol (4).

### 1.1. Alcohol use disorder and the major issue contributed by alcohol craving

One of the commonest negative impacts of alcohol consumption is the occurrence of alcohol use disorder. Alcohol use disorder is a chronic and recurrent mental disorder (5). It will affect the brain's ability to control emotions, decisions, and behaviors, resulting in the decline of work and learning abilities, impaired physical and mental health, and even legal problems (5). Alcohol use disorder poses a serious threat to individual health, family wellbeing, and social stability (6). Therefore, how to effectively treat the disease and prevent relapse is of great significance.

The core symptoms of alcohol use disorder include two aspects: physical dependence based on increased alcohol tolerance and withdrawal reaction and psychological dependence based on alcohol craving (5, 6). Currently, the clinical treatment for alcohol use disorder is focused on reducing physical dependence, and in fact, this is the only effective treatment available for alcohol use disorder. Treatment methods for physical dependence mainly include the prescription of benzodiazepines which is administered to alleviate withdrawal symptoms, low-dose antipsychotic to relieve psychotic symptoms associated with alcohol withdrawal, and adequate vitamin B6 supplementation to prevent Wernicke encephalopathy. However, most patients remain psychologically dependent on alcohol during abstinence, and their hazardous drinking persists due to the satisfaction and euphoria contributed by persistent drinking, resulting from strong alcohol craving (7), even after receiving standardized in-patient treatment. This psychological dependence on alcohol is hard to resist, so it is extremely difficult to maintain long-term abstinence, and hence, alcohol use disorder is prone to relapse (5). Since psychological dependence is the key factor leading to relapse of hazardous alcohol use after abstinence, it is essential to monitor and manage alcohol craving to improve the therapeutic outcome of alcohol use disorder.

### 1.2. Exploration of electrophysiological monitoring technology based on electroencephalogram (EEG) in alcohol use disorder

In recent years, the application of electrophysiological technology to explore the mechanism of addiction diseases has become an important research interest. Electrophysiological monitoring methods based on EEG have made a lot of progress in the diagnosis and treatment of alcohol use disorder, including resting EEG and task-state EEG, and the latter is mainly event-related potentials (ERP) and event-related oscillations (ERO) (6). EEG-based monitoring technology can reflect neuropsychological factors from different perspectives, quantify brain energy fluctuations under specific mental activities to a certain extent (8), and help judge the severity of alcohol use disorder.

ERP is an objective detection tool to study psychological activities, which can reflect the cognitive process through the EEG activities under task state (8, 9). ERP has very high temporal resolution, which can reflect the dynamic balance between excitation and inhibition of brain neural network within milliseconds (9), so it is suitable for studying rapidly changing mental activities. Moreover, the detection equipment is convenient and can overcome small movement or noise, which is widely applied to various subjects. A previous study suggested that the abnormal amplitude of ERP might be a disease marker of substance dependence or a potential neurobiological endophenotype of addiction (10). Another study showed that alcohol users had enhanced ERP response to alcohol-related cues, especially in male patients (11). A previous study by our team found that there were positive results in EEG analysis of alcohol-dependent patients in craving state evoked by static pictures with alcohol cues (12). However, the alcohol-dependent group showed lower N200 amplitude and higher P300 amplitude under the stimulation of alcohol cues and was positively correlated with the scores of subjective craving scales. Alcohol cue reactivity was used to evoke alcohol craving in patients with alcohol use disorder, and this ERP was monitored at the same time. In this way, it was possible to objectively assess the psychological activities associated with alcohol craving.

### 1.3. Application of virtual reality technology in alcohol addiction

Virtual reality (VR) is a computer simulation technology, which synthesizes various computer functions to generate a realistic virtual environment (VE). In the digital world created by VR-related devices, users can get an “immersive” experience across time and space constraints, and the VE with multimodal stimuli can be customized and changed according to user needs (13). In recent years, VR technology has been increasingly applied in the medical field and has been well-developed in psychological rehabilitation, especially in the treatment of anxiety disorder and post-traumatic stress disorder (14). VR therapy, as an intervention method with flexible treatment time, low cost, and personalized scheme, should be applicable to the treatment of a variety of mental diseases. At present, the application of VR technology in alcohol use disorder is still in the early stage of development and implementation.

Some pieces of research try to integrate VR into the psychotherapy of alcohol use disorder and have obtained new discoveries and understandings. Cue exposure therapy (CET) aims to reduce cue reactivity through repeated exposure to substance-related cues, leading to habituation, so as to reduce alcohol craving (15). However, a meta-analysis pointed out that the efficacy of traditional CET in the treatment of alcohol use disorder was limited or even not better than CBT (16), which may be associated with the failure to fully simulate the real exposure environment in many CET cases. In view of this situation, researchers have tried virtual reality exposure therapy (VRET). VR technology can simulate realistic exposure situations to improve the factors that limit CET efficacy. VRET provides some immersive three-dimensional environments and social interactions similar to the real world, and it treats diseases in a novel and attractive way (17). Bordnick et al. (18) found that alcohol cues VE evoked higher subjective craving in alcoholics than neutral cues VE, and patients paid more attention to the alcohol cues. In addition, subjects reported a good sense of presence (18). Cho et al. pointed out that the situation with social pressure provided by virtual characteristics can evoke a higher degree of alcohol craving than the situation without social pressure, and they believed that the social pressure provided by VR was more important than mere alcohol cues (19). There is reason to believe that the more complex, real-life cue exposure scenario provided by VR is conducive to sufficiently evoking craving, so as to implement better VRET.

### 1.4. Application of acceptance and commitment therapy in addiction medicine

ACT is a third-generation cognitive behavioral approach that uses acceptance and mindfulness processes, as well as commitment and behavior change processes to produce psychological flexibility (20). ACT was designed to increase adaptive coping through acceptance, cognitive defusion, mindfulness, and perspective-taking exercises while supporting clients in aligning behavior with their personal values (21). The objectives of the therapy are for clients to learn that avoidance, suppression, or attempts to control difficult thoughts can be counterproductive. Clients also learn to focus on behaviors and actions that are in line with their individual values (22, 23).

A meta-analysis reported that ACT showed better efficacy compared with other therapies when applied to substance use disorders (SUDs), including tobacco, alcohol, and illicit drug use (24). A study comparing the efficacy of ACT and cognitive behavioral therapy (CBT) in treating SUD found that ACT was better than CBT in reducing drug use and improving mental health (25). Therefore, in the long term, ACT may be more appropriate than CBT for treating substance addiction. Another study on male smokers showed that ACT significantly improved smoking cessation rate and alleviated comorbid depression and anxiety symptoms (26). With the promising potential of ACT in the treatment of emotional disorders and SUD, we are interested in whether ACT can more specifically be used for treating alcohol use disorder. A systematic review suggested that ACT provides an alternative to existing treatments for alcohol use disorder, with promising results, but further research is needed (27). Witkiewitz et al. (28) pointed out that alcohol addiction is actually a fixation on avoiding the individual's cognitive, emotional, and social experiences, especially the craving for alcohol and negative emotions. The key to break the vicious cycle of addiction is to make clients accept their negative emotions and internal pain and reduce their experiential avoidance. Mindfulness, which emphasizes openness and acceptance, that can help patients with alcohol use disorder accept their negative emotional experience and drinking craving, thus improving the curative effect (28). In a systematic review of studies on the efficacy of VRET for substance dependence, only five studies were selected for review which investigated VRET efficacy for the treatment of alcohol craving (one study) and nicotine craving and dependence (four studies). The review reported that VRET was effective in reducing nicotine craving and facilitating smoking cessation. In addition, the review also indicated that VRET was more effective than cognitive behavioral therapy to alleviate alcohol craving, but the selected study was limited by small sample size and short period of follow-up (29).

### 1.5. Rationale of the study

Since alcohol craving is a major factor leading to disease relapse in patients with alcohol use disorder (5, 7), it is essential to focus on the monitoring and intervention of craving to improve efficacy. However, the following problems exist in clinical practice: first, there is a lack of objective methods to detect psychological dependence on alcohol in clinic, and alcohol craving can only be quantified subjectively through psychological rating scales. Second, the treatment of alcohol use disorder, especially the effective intervention aimed at treating psychological craving, is lacking, and patients are prone to relapse.

Hence, it is important to explore an objective detection method for alcohol craving. Medical detection technology based on electroencephalogram (EEG) can reflect neuropsychological factors from different perspectives and can also quantify brain energy fluctuations under specific psychological activities to a certain extent (8). Monitoring event-related potential (ERP) of alcohol craving evoked by alcohol cues, which are a type of task-state EEG, may be helpful for objectively evaluating the psychological activities associated with craving.

In view of the current difficulties in treating alcohol use disorder (5, 7, 30, 31), it is of great significance to investigate the effects of new treatment methods or adjuvant therapy. Acceptance and commitment therapy (ACT) facilitates the development and maintenance of health behavioral improvements (20). With the promising potential of ACT in the treatment of affective disorders and substance use disorders (SUDs), it may be promising to treat alcohol use disorder. In addition, virtual reality exposure therapy (VRET), an innovative therapy technique utilizing computer virtual reality technology that treats mental disorders well, is also worthy of evaluating its efficacy for use in the treatment of alcohol use disorder.

### 1.6. Objectives

#### 1.6.1. Primary objectives

The primary objective was to evaluate the efficacy of virtual reality exposure therapy (VRET) to ameliorate alcohol craving as compared with acceptance and commitment therapy (ACT) and treatment as usual across four timelines, such as t_0_ (pre-intervention), t_1_ = 4 weeks (immediately after the end of intervention), t_2_ = 12 weeks (8 weeks after the end of intervention), and t_3_ = 24 weeks (20 weeks after the end of intervention).

#### 1.6.2. Secondary objectives

The secondary objectives were as follows:

- To evaluate the efficacy of virtual reality exposure therapy (VRET) to ameliorate the severity of alcohol use disorder as compared with acceptance and commitment therapy (ACT) and treatment as usual across four timelines, such as t_0_ (pre-intervention), t_1_ = 4 weeks (immediately after the end of intervention), t_2_ = 12 weeks (8 weeks after the end of intervention), and t_3_ = 24 weeks (20 weeks after the end of intervention).
- To assess the efficacy of virtual reality exposure therapy (VRET) to ameliorate comorbid depression and anxiety symptoms as compared with acceptance and commitment therapy (ACT) and treatment as usual across four timelines, such as t_0_ (pre-intervention), t_1_ = 4 weeks (immediately after the end of intervention), t_2_ = 12 weeks (8 weeks after the end of intervention), and t_3_ = 24 weeks (20 weeks after the end of intervention).
- To assess the efficacy of virtual reality exposure therapy (VRET) to normalize event-related potential (ERP) in electroencephalogram (EEG) as compared with acceptance and commitment therapy (ACT) and treatment as usual across four timelines, such as t_0_ (pre-intervention), t_1_ = 4 weeks (immediately after the end of intervention), t_2_ = 12 weeks (8 weeks after the end of intervention), and t_3_ = 24 weeks (20 weeks after the end of intervention).

## 2. Methods and analysis

### 2.1. Study design and setting

This multicenter, three-armed, parallel, single-blind randomized controlled trial is expected to run for a duration of 3 years (from May 2023 to April 2026). The proposed study site for subject recruitment is the Second Affiliated Hospital of Xinxiang Medical University, Henan, China, and Advanced Medical and Dental Institute, Universiti Sains Malaysia.

### 2.2. Sample size

The estimated sample size will be calculated using the G^*^Power 3.1.9.7 sample size calculator, using the sample size calculation for repeated measure ANOVA and within–between interaction, whereby the type I error was 0.05, the power was 0.8, the number of groups was three (VRET, ACT, and TAU groups), the number of measures was five (degree of alcohol craving, severity of alcohol use disorder, depression symptoms, anxiety symptoms, and changes in ERP), and the effect size was 0.13, which was cited from a randomized clinical trial that compared the efficacy of virtual reality therapy with multisensory stimulation and cognitive behavioral therapy in alleviating alcohol craving in alcohol dependence patients (32). The estimated total sample size needed was 120 (after inclusion of 30% dropout), in which 40 subjects per group are required.

### 2.3. Sampling method

The sampling method used in this study for the recruitment of participants is by consecutive sampling.

### 2.4. Recruitment of subjects

The source population comprised of male patients diagnosed with alcohol use disorder (AUD) [only male patients are selected as the prevalence of AUD is much higher in men than in women and the majority of patients presenting to the Addiction out-patient clinics are men (5)] registered with the Addiction Outpatient Clinic, Second Affiliated Hospital of Xinxiang Medical University and the Psychiatry Outpatient Clinic, Advanced Medical and Dental Institute, Universiti Sains Malaysia. All potential patients will be approached by the research assistant and provided with brochures that provide information on the research project, the intervention program, duration of the study, and sample collection, as well as a feedback form to enable the research team to identify patients interested to participate in the study. The research assistant will explain the contents of the brochures to the potential patients to ensure that they understand and are clear with the information about the study. Patients who voluntarily agreed to participate in the study will be screened for inclusion and exclusion criteria.

The inclusion criteria are as follows: (1) hospitalized patients diagnosed with alcohol use disorder (confirmed by the relevant diagnostic criteria of Diagnostic and Statistical Manual of Mental Disorders-V); (2) male patients, aged 18–55 years old, Han nationality in China or Malaysian Chinese, junior high school education or above, right-handed (because of the difference in EEG between right-handed and left-handed persons); and (3) those with normal eyesight (including corrected vision). The exclusion criteria are as follows: (1) those with current and lifetime history of abuse of any other psychoactive substances (except tobacco); (2) those with current and lifetime history of mental disorders; (3) those with current and lifetime history of central nervous system diseases or serious medical illnesses; (4) those who are unable to complete the EEG detection or psychological scale assessment; and (5) those who have been administered with psychotherapy or VRET in the past.

Those who are eligible will be offered to participate in the study and explained the objectives and procedures of the study, benefits and risks of participation, their rights to withdraw, anonymity of data collected, data storage, and the incentives, before they signed the informed consent. Consent will be obtained from the research assistant who is not involved in the study. To ensure sufficient participants are recruited for the study, recruitment advertisement regarding the study will be posted in the Second Affiliated Hospital, Xinxiang Medical University and Advanced Medical and Dental Institute, Universiti Sains Malaysia, and the participants will receive an honorarium to compensate for their time spend and travel expenses to participate in this study.

### 2.5. Randomization

The randomization method used for this study is stratified permuted block randomization, in which the trial participants are stratified according to marital status (the strata selected are married, single, and divorced/widower/separated) and monthly income (the strata are <RMB 5,000, RMB 5,000 to RM 10,000, and >RMB 10,000). The participants will be randomized into three groups, such as the VRET group, the ACT group, and the treatment as usual (TAU) group, in a 1:1:1 ratio by a research assistant who is not involved in the study and who is not aware of the study objectives. The randomization is computer generated in which the allocation number is concealed in an opaque envelope that will be given to the participants. Randomization will be carried out by a research assistant who is not involved in the study and does not know the objectives of the study. The research assistant will receive some training on the randomization procedures before the process begins.

### 2.6. Intervention

Initially, participants will be admitted to the in-patient ward at the Second Affiliated Hospital, Xinxiang Medical University and at Advanced Medical and Dental Institute, Universiti Sains Malaysia, for conventional treatment of detoxification to treat alcohol withdrawal on abstinence from alcohol. The treatment regimen for detoxification includes benzodiazepine replacement therapy, low dose of antipsychotic, thiamine (vitamin B6), and medication for symptomatic relief. The conventional detoxification will continue for a duration of 2 weeks in the ward, to ensure that participants abstain from alcohol use for at least 2 weeks.

Then, all subjects will be randomly assigned to three groups: (i) patients will be provided with ACT for 4 weeks in combination with conventional treatment, (ii) patients will be provided with VRET for 4 weeks in combination with conventional treatment, and (iii) patients receiving only conventional treatment and non-specific ingredients of the psychotherapeutic approach for 4 weeks.

The ACT intervention will be conducted individually. The ACT modules cover eight sessions, 1 h in each session. The sessions will be held twice a week for 4 weeks (33, 34). The VRET intervention will be conducted in a single patient for each session. The total course of VRET includes 12 sessions, 25 min in each session. The sessions will be held three times a week; hence, a total duration of 4 weeks is needed (32). In the control group, each participant will receive eight sessions on non-therapeutic approaches (each session is 1 h), twice a week for 4 weeks.

#### 2.6.1. Virtual reality exposure therapy

Each session of VRET will last for 25 min and consists of three parts: 5 min of relaxation, 10 min of exposure to high-risk situation, and 10 min of exposure to aversive situation. The relaxation scene includes four beautiful landscapes, and patients can choose any of these four, as perceived to be the most comfortable landscape. The visual stimulation of high-risk scene is designed as any combination of four different scenes (street barbecue stands, restaurant, bar, and home) and four types of alcoholic beverages (Chinese liquor, beer, grape wine, and cocktail), which are customized according to the patient's personal preference. At the same time, the odor of the alcoholic beverage of the patient's choice will be provided as an olfactory stimulation. The aversive situations are visual and auditory stimuli provided by a series of VR videos depicting the harmful effects of alcohol consumption. The VRET session course will be implemented with Oculus Quest VR equipment, whose head screen has a single-eye resolution of 1,600 × 1,440 and field of view of ~100 degrees. It supports the access of left and right dual-channel earphones, providing excellent visual and auditory sensory experience. The VRET manual for this study is presented in Appendix 1.

#### 2.6.2. Acceptance and commitment therapy

The ACT intervention consists of eight sessions, twice a week for 4 weeks. The initial session began with building a therapeutic rapport with the participant and gathering personal information on cognitive fusion, external barrier, unworkable action on alcohol use, experiential avoidance, personal strength, support network, and past life events. Based on the information, a case formulation is constructed. Subsequent sessions involved learning on acceptance and acknowledgment of unpleasant thoughts and feelings associated with alcohol craving and clinical features of alcohol use disorder, cognitive defusion on how to dissociate self-avoidance of unpleasant thoughts and feelings, learning and practicing mindfulness exercise (such as mindfulness breathing) to make self-aware of the many things that occurred presently which can be enjoyed and focus on rather than focusing on unpleasant thoughts and feelings, and learning to be aware of the safe self which is separated from the unpleasant thoughts and feelings and identifying values in life and strategies to achieve these values (therapeutic processes in hexaflex). The ACT manual for this study is included in Appendix 2.

#### 2.6.3. Treatment as usual control

Each participant will receive eight sessions of non-therapeutic activities, such as understanding the psychological indication of treatment, identification of current problems, and opportunity for disclosure and reassurance. The amount of time spend with a professional facilitator for each session is the same as for the other two groups, whereby each session lasted for 1 h and held twice a week for 4 weeks. The participants will be allowed to select either ACT or VRET treatment after the completion of the study.

### 2.7. Treatment fidelity

The ACT sessions will be conducted by four trained therapists who are postgraduate students in psychology and who had experience in conducting psychotherapy for the past 2 years. Initially, these selected postgraduate students will be enrolled in a 2-day workshop on ACT training which is conducted by the principal investigator (PI). Then, they will participate in ACT sessions on alcohol use disorder patients in the out-patient clinic of the PI (from Universiti Sains Malaysia) and co-PI (from Xinxiang Medical University) for 1 month in order to be trained for ACT. For the assessment of the integrity of the ACT sessions conducted, one psychiatrist and one clinical psychologist trained in ACT will conduct the assessments. Then, 15% of the audio-visual recording of the ACT sessions of the four therapists will be randomly selected following stratification with regard to the therapist (first, second, third, and fourth therapists) and the phase of the intervention (early, mid, and late phases of the therapy sessions). The assessment will be performed using the Drexel University CT/ACT Therapist Adherence and Competence Rating Scale (DUACRS) (35). The first assessor will evaluate 50% of the selected recording, and the second assessor will assess another 50% of the recording. Then, the inter-rater reliability between the two assessors will be computed to rate the integrity of the ACT sessions by all the therapists. In addition, any therapists who have any issues while conducting the ACT sessions will discuss their issues with the principal investigator.

### 2.8. Blinding

The researchers will be blinded in this study, in which the recruitment and randomization of the participants will be conducted by a research assistant not involved in this study. The data collection will also be carried out by another research assistant who is also not involved in the study and does not know the objectives of the study. A statistical analysis plan will also be prepared, and the statisticians who will analyze the data are also not part of the research team. The researchers will only be unblinded after the data analysis is completed. The researchers will be unblinded if serious adverse effects occurred in the participant which may or may not be related to the study intervention. The participants are not blinded due to significant differences in the features of the intervention group.

### 2.9. Measures

This study follows the Standard Protocol Items: Recommendations for Intervention Trials (SPIRIT) 2013. The checklist for SPIRIT is included in Appendix 3. The schedule for assessments in this study is summarized in Table 1. There will be four timelines of assessment. Baseline assessment (t_0_) commences after the randomization of participants into the three intervention groups (VRET, ACT, and control groups), wherein the sociodemographic, clinical, and alcohol use characteristic questionnaire, Alcohol Use Disorder Identification Test (AUDIT), Penn Alcohol Craving Scale (PACS), Hamilton Anxiety Rating Scale (HAM-A), and Hamilton Depression Rating Scale (HAM-D) are administered, while event-related potential (ERP) detection in electroencephalogram (EEG) is performed in the participants. Then, during follow-up assessment, such as t_1_ = 4 weeks (immediately after completion of intervention), t_2_ = 12 weeks (8 weeks after completion of intervention), and t_3_ = 24 weeks (20 weeks after completion of intervention), the questionnaires are re-administered to the participants which include alcohol use characteristics, AUDIT, PACS, HAM-D, and HAM-A, while ERP detection in EEG is re-assessed. The serial assessments will be carried out by a research assistant who is not involved in the research project and does not know about the objectives of the study. The research assistant will receive some training on the administration of the measures before the data collection begins. To ensure that the participants complete the serial assessments and interventions, an appointment card is given to each participant with the next appointment date written on the card. Emails and messages will be distributed 3 days prior to the appointment to remind the participants regarding the appointment date, and if any participant does not reply, a phone call will be made a day before the appointment date.

**Table 1:** Schedule of assessments of the study outcome measures.

	**Enrollment**	**Allocation**	**Post-allocation**		
**Time**	***–t**_1_*	***t**_0_* **(0 week)**	***t**_1_* **(4 weeks)**	***t**_2_* **(12 weeks)**	***t**_3_* **(24 weeks)**
**Enrollment:**					
Eligibility screen	X				
Informed consent	X				
Allocation		X			
**Interventions:**					
(1) Virtual reality exposure therapy (VRET)			X		
(2) Acceptance and commitment therapy (ACT)			X		
(3) Controls (treatment as usual)			X		
**Assessments:**					
(1) (a) Sociodemographic and clinical characteristics data		X			
(b) Alcohol use characteristics (number of drinks per drinking days, number of hazardous drinks per week or per drinking day, and frequency of binge drinking in previous week)		X	X	X	X
**(2) Primary outcomes:**					
(a) Penn Alcohol Craving Scale (PACS)		X	X	X	X
**(3) Secondary outcomes:**					
(a) Alcohol Use Disorder Identification Test (AUDIT)		**X**	**X**	**X**	**X**
(b) Hamilton Anxiety Rating Scale (HAM-A)		X	X	X	X
(c) Hamilton Depression Rating Scale (HAM-D)		X	X	X	X
(d) event-related potential (ERP) detection in electroencephalogram (EEG)		X	X	X	X

#### 2.9.1. Sociodemographic, clinical, and alcohol use characteristics

The sociodemographic and clinical characteristics recorded from the participants include age, marital status, monthly household income, occupation status, education attainment, history of cigarette smoking, current and lifetime history of medical illness, current and lifetime history of psychiatric illness, and current medication history.

The alcohol use characteristics recorded include duration of alcohol use, average number of drinks during drinking days, frequency of binge drinking in the previous week (binge drinking is defined as five drinks on an occasion in men and four drinks on an occasion for women) (36), and the number of hazardous drinks per day or per week (hazardous drinking is defined as 14 drinks per week or four drinks per drinking day for men and seven drinks per week or three drinks per drinking days for women) (37).

#### 2.9.2. Primary outcome (degree of alcohol craving)

##### 2.9.2.1. Penn Alcohol Craving Scale

This self-administered instrument is used to measure the degree of alcohol craving among alcohol users. It consists of five items in a single factor. Each item is scored on a Likert scale ranging from 0 (none) to 6 (extremely severe). The items are designed to rate the frequency, duration, intensity, average craving degree, and difficulty to cope with craving experienced in the past week. The higher the score, the more severe the degree of alcohol craving (38). The instrument is translated and validated in the Chinese alcohol user population and exhibits an excellent internal consistency with Cronbach's α of 0.97 (39).

#### 2.9.3. Secondary outcomes

##### 2.9.3.1. Alcohol Use Disorder Identification Test

This self-administered tool is used to assess the severity of alcohol use disorder among alcohol users. The tool consists of 10 items and three domains, in which three items are designated to the amount and frequency of the alcohol consumption domain, three items are assigned to alcohol dependence domain, and another four items are allocated to various problems caused by the alcohol domain. It has a cutoff score of ≥8 indicating the presence of alcohol use disorder. A high score in the first three items and a low score in the other items indicate serious harmful drinking, while a high score in items 4, 5, and 6 denotes a significant degree of alcohol dependence, and a high score in the last four items demonstrate hazardous drinking (40). The tool was translated and validated in the Chinese alcohol use disorder population, and its internal consistency was acceptable with Cronbach's α of 0.782 (41).

##### 2.9.3.2. Hamilton Depression Rating Scale

The HAM-D is an observer-administered instrument to assess the severity of depressive symptoms. The HAM-D consists of 21 items but is scored on the first 17 items. The higher the score, the greater the severity of depressive symptoms. The interpretation of the total score is such that the score of 0–7 indicates normal, 8–16 denotes mild depression, 17–23 indicates moderate depression, and 24 and above denotes severe depression (42). The instrument is translated and validated in the Chinese population with an acceptable internal consistency with Cronbach's α of 0.714 (43).

##### 2.9.3.3. Hamilton Anxiety Rating Scale

The HAM-A is an observer-administered instrument to assess the severity of anxiety symptoms. It consists of 14 items which assess 14 anxiety symptoms. Each item is scored on a Likert scale ranging from 0 (not present) to 4 (severe). A total score of <17 indicates mild severity, 18–24 denotes mild to moderate severity, 25–30 indicates moderate to severe, and >30 indicates severe (44). The instrument is translated and validated in the Chinese population with an excellent internal consistency with Cronbach's α of 0.93 (45).

##### 2.9.3.4. Event-response potential

###### 2.9.3.4.1. Experimental paradigm.

Visual stimuli will be presented by E-Prime 2.0 software. There are three types of visual stimuli, such as (a) images related to alcohol cues (for example, common places where alcohol is consumed, habitual alcohol products, and people drinking alcohol), (b) neutral images which are not related to alcohol cues, and (c) task-related images which require key operation. The EEG monitoring and recording will be carried out, while the participants watch the stimulation paradigm.

###### 2.9.3.4.2. Acquisition of EEG signal.

EEG signal monitoring and recording are performed using the Brain Amp MR-32 instrument and Brian Vision Recorder software. A total of 64 scalp positions will be recorded by using the 10-10 standard lead system. The sampling rate will be adjusted to 1,000 Hz, and the impedance between the electrodes and the skin of the scalp will be < 5 kΩ.

###### 2.9.3.4.3. ERP analysis.

The data analysis for ERP will be carried out using Brian Vision Analyzer 2.1 software. The processes involved are re-reference, filtering, removal of ocular artifacts, artifact removal, segmentation, baseline correction, and peak detection.

##### 2.9.3.5. Other measures

An electronic diary will be created which allows the participants to record their progress in doing home assignments between each ACT session, to ensure that the practice of ACT strategies learned in each session is consolidated. In addition, any dropout from the study and its reasoning (lack of interest to continue, being ill, transportation, and logistic issues) will also be recorded.

### 2.10. Data analysis

Data analysis will be carried out using the Statistical Package for Social Sciences version 26 (SPSS-26, Inc., Chicago, IL, USA). Descriptive statistics for sociodemographic, clinical, and alcohol use characteristics, AUDIT, PACS, HAM-D, and HAM-A scores, and ERP will be computed. For nominal and ordinal data, these will be presented in frequency and percentage. For continuous data, it will be presented as mean and standard deviation if the variable is normally distributed. While for non-normally distributed data, it will be presented as median and interquartile range. The normality of the data will be determined by Shapiro–Wilk test.

As for the assessment of the primary outcome, one-way ANOVA with false discovery rate adjustment is used to compare the mean of the outcomes (PACS, average number of drinks during drinking days, frequency of binge drinking in the previous week, and the number of hazardous drinks per day or per week) between the three groups (VRET, ACT, and control groups) at each specific time point (t_0_, t_1_, t_2_, and t_3_). While for the main analysis, mixed ANOVA is used to evaluate the interaction of intervention and time on the outcomes will be computed (outcomes: PACS, average number of drinks during drinking days, frequency of binge drinking in the previous week, and the number of hazardous drinks per day or per week; interaction: intervention × time; whereby time is the within-subject factor, while intervention is the between-subject factor). The main effect of the interaction will be presented as estimated marginal mean and standard error of mean. The primary outcome assessment will follow the intent-to-treat principle. The data analysis for the secondary outcomes (AUDIT, HAM-D, and HAM-A scores and ERP) will be analyzed in the same way as the primary outcomes. Statistical significance is set at *p* < 0.05 and is two-tailed.

As for the management of missing data, if there is only 5% of missing data or less, it will be ignored and data analysis will proceed. However, if the missing data are from 5 to 40% and are missing randomly, multiple imputations with restricted maximum likelihood estimates will be performed using Stata 15, to estimate the missing values. However, if the missing data are more than 40% or are completely randomly missing or non-randomly missing, data analysis of the remaining data will proceed, and the missing data issue will be reported under the limitation of the study (46).

### 2.11. Data availability

Anonymous data will be made available after the publication of the findings of the study. The data will be uploaded to the Figshare data repository after the completion of the study.

## 3. Discussion

This study primarily investigates the efficacy of VRET compared with ACT and treatment as usual to depreciate the degree of alcohol craving for people with alcohol use disorder to reduce relapse. In addition, this study also evaluates the efficacy of VRET compared with ACT and treatment as usual to dissipate the severity of alcohol use disorder, and comorbid depressive and anxiety symptoms and normalize the ERP among the alcohol use disorder patients across four timelines (from the baseline to immediately after completion of intervention, 8 weeks after completion, and 20 weeks after completion). The research findings will provide evidence to treating clinicians regarding efficacious psychotherapy or psychosocial intervention for the treatment of alcohol use disorder to reduce the rate of relapse, in which currently available pharmacotherapy has little success. Moreover, if VRET and ACT are proven efficacious to treat alcohol craving, these interventions should also be investigated for their efficacy to treat craving and prevent relapse in other substance use disorders in the near future, whereby the methodology of this RCT could be replicated in future studies.

There are a few limitations to be considered in this study. First, this study only recruits male alcohol use disorder patients, which may limit the generalizability of the study findings as it may not be representative of the entire alcohol use disorder population in China and Malaysia. Second, this study will only recruit subjects with Han ethnicity in China and Malaysian Chinese, which may not be representative of the alcohol use disorder population in China and Malaysia as both countries' populations consist of multi-ethnic background. Finally, the differences in the scheduling of interventions and duration of the session between the VRET and ACT groups may lead to differences in findings due to discrepancies in the settings rather than due to differences in efficacy between the two interventions. However, we attempted to align the scheduling of the sessions in all the three groups as similarly as possible. VRET is scheduled for 25 min/session, five times a week for 20 sessions (total duration = 4 weeks), ACT will be conducted for 1 h/session, two times a week for eight sessions (total duration = 4 weeks), and the control group session is performed at 1 h/session, two times a week for eight sessions (total duration = 4 weeks).

## 4. Ethics and dissemination

This study was approved by the Human Research Ethics Committee of Xinxiang Medical University [code: XYEFYLL-(Research)-2022-29] and the Human Research Ethics Committee of Universiti Sains Malaysia (code: USM/JEPeM/22080545). This study will abide by the ethical conduct of the Declaration of Helsinki 1964 and its subsequent amendments and also the Clinical Good Practice guidelines for clinical trial in Malaysia. The English and Chinese versions of the participant information sheet and consent form are included in Appendices 4, 5. A clinical trial monitoring committee will be set up which will be led by the principal investigator in coordination with the trial coordinator and field coordinator and will meet up every week to discuss, record, and report the day-to-day conduct of the clinical trial and audit of the trial which will be submitted to the human research ethics committee of both Xinxiang Medical University and Universiti Sains Malaysia. Auditing of the trial will be carried out by the Clinical Trial Coordination Unit of the Second Affiliated Hospital of Xinxiang Medical University and Advanced Medical and Dental Institute of Universiti Sains Malaysia and independent from the funder of the RCT. If there are any changes in the initial research protocol submitted (such as changes in the subject recruitment, data collection, and data analysis.), the changes will be reported to the Human Research Ethics Committee of both Xinxiang Medical University and Universiti Sains Malaysia.

The following information will be delivered to all participants before they are enrolled in the study:

- The right of the participant to withdraw from the study at any time without the need to provide any reason and the data collected from them will be discarded and will not be used for the study.
- The anonymity of the participant is assured, and no personally identifiable information will be used for research purposes. For example, all participants will be provided with a research number for identification purposes, such as VT001 and VT002. The group data will be computed and reported as research findings instead of using individual data.
- The data collected will be stored in a research file which will be locked in a file cabinet where the key will be kept by the principal investigator. In addition, the data collected may also be stored in thumb drive which will be kept by the principal investigator. Only the research team members have access to the research file and thumb drive.
- The collected data may be stored for a duration of at least 2 years after completion of the study, and then, it will be discarded following the standard procedures. Any reuse of the data will need consent from the participants.
- The research team will only access the collected data for publication purposes and presentation in academic conferences or symposia. As for publication of the research findings, the principal investigator is the corresponding author while the other author arrangement will be decided based on the International Committee of Medical Journal Editors' recommendations. All the researchers declared that there is no competing interest in any financial gain or conduct of the study.

Adverse effect is any untoward event that occurred in relation to or not related to the interventions in the clinical trial. All participants will be given a clinical trial card which contains the full name, telephone number, and email of the person in charge of adverse effect reporting in the research team. All participants who experience any adverse effects are encouraged to contact the person in charge of the research team. All adverse effects have to be reported in the adverse effect section of the case report form, in which the following details need to be filled, such as the date and time of adverse effects occurrence, the duration, description of the adverse effects, treatment seek, time taken for adverse effects to resolve or still on-going. Participants who experience adverse effects will be withdrawn from the study. The events are considered adverse effects which lead to the withdrawal of participants from the study include the following:

- Any adverse effect or reaction which is unrelated to the intervention under investigation in the study;
- Any adverse effect or reaction which is related to the intervention under investigation in the study, which occurred after the intervention is administered;
- Any adverse effect or reaction which leads to severe psychological distress, psychotic symptoms, change of behavior and personality, deliberate self-harm, or suicidal tendency;
- Any adverse effect or reaction which is suspected or unexpectedly associated with the intervention under investigation according to conventional practice.

All adverse effects need to be reported to the Human Research Ethics Committee of Xinxiang Medical University or Universiti Sains Malaysia. An interim analysis will be conducted to investigate the adverse effect reported in a clinical trial and if found to induce risk of morbidity or mortality and it is related to the intervention under investigation; then, the clinical trial will be terminated prematurely. Adverse effects which will lead to premature termination of the clinical trial include those that induce deliberate self-harm, suicidal tendency, and hospitalization of the participants. As for participants who reported serious adverse effects during post-trial, they will be referred to the nearest treatment facility for further treatment, and they will also be compensated if the serious adverse effect is related to the study intervention.

## Ethics statement

The studies involving human participants were reviewed and approved by (1) Human Research Ethics Committee USM, Division of Research and Innovation (R&I), USM Health Campus, 16150, Kubang Kerian, Kelantan. (2) Human Research Ethics Committee Xinxiang Medical University, Second Affiliated Hospital, Xinxiang Medical University, 453002 Xinxiang, Henan, China. The patients/participants provided their written informed consent to participate in this study.

## Author contributions

ML and CW conceptualized the study. HD, RZ, CW, JW, SW, JZ, and ML contributed to the investigation and methodology of the study. RZ and CW obtained funding for the study and contributed to manage resources. HD, RZ, CW, JW, SW, JZ, ML, and NS contributed to visualization, software, validation, and interpretation of the study. ML, RZ, CW, and NS supervised the study. HD and ML wrote the first draft of the manuscript. HD, RZ, CW, NS, JW, SW, JZ, and ML revised and approved the manuscript for submission. All authors contributed to the article and approved the submitted version.
