# Supplementary material for: The efficacy of virtual reality exposure therapy for the treatment of alcohol use disorder among adult males: a randomized controlled trial comparing with acceptance and commitment therapy and treatment as usual
Source: Front Psychiatry. 2023 Aug 22;14:1215963. doi: 10.3389/fpsyt.2023.1215963 (PMC10477784; doi:10.3389/fpsyt.2023.1215963)
Supplement: Supplementary file 1 [file Data_Sheet_1.docx]

**Supplementary appendix 1. The virtual reality exposure therapy module for this study (Lee et al., 2007; Lee et al., 2009):**

The virtual reality exposure therapy (VRET) module:

The entire VRET course lasts 4 weeks, with a total of 12 sessions, each lasting 25 minutes. Two psychiatrists will be present during each treatment session to supervise and assist. Patients will participate in a 5-minute VR orientation in a non-study related environment to familiarize themselves with the VR experience, input device operation, and trial procedures prior to their initial VR treatment.

Each VRET session consists of three parts: first the relaxation scene, then the high-risk scene, and finally the aversion scene.

Relaxation scene: The initial relaxation scene includes a choice of four different beautiful landscapes, from which patients can choose the one they feel most comfortable experiencing. This scenario was set up to allow patients to quickly adapt to the VR experience, gain excellent immersion, and enter treatment in a comfortable and relaxed manner.

High-risk scene: The subsequent high-risk scene settings are customized to each patient's personal preferences. The stimulus scenario consisted of four different drinking venues (street barbecue stands, restaurant, bar and at home) and four types of alcoholic beverages (Chinese liquor, beer, grape wine and cocktail) in any combination to provide visual stimulation. At the same time, an olfactory stimulation is provided by cotton balls soaked in the chosen alcoholic beverage. High-risk scene provides an immersive interactive experience where patients are repeatedly exposed to highly realistic drinking craving-evoking cues that are gradually tolerated and adapted to achieve desensitization.

Aversion scene: The aversion scene includes visual and auditory stimuli. Patients will watch a series of VR video depicting the harmful effects of alcohol consumption: vomiting after drinking, alcoholic delirium tremens, car accidents after drinking, and arguing with family members after drinking. Through this form of aversion therapy, patients are made to feel the seriousness and harmful effects of alcohol addiction to themselves, their families and the society, and are guided to reject alcohol psychologically.

**References**

(1) Lee JH, Kwon H, Choi J, Yang BH. Cue-exposure therapy to decrease alcohol craving in virtual environment. *Cyberpsychol Behav.* (2007) 10(5):617-623. doi:10.1089/cpb.2007.9978.

(2) Lee SH, Han DH, Oh S, et al. Quantitative electroencephalographic (qEEG) correlates of craving during virtual reality therapy in alcohol-dependent patients. *Pharmacol Biochem Behav.* (2009) 91(3):393-397. doi:10.1016/j.pbb.2008.08.014.
