# Supplementary material for: The efficacy of virtual reality exposure therapy for the treatment of alcohol use disorder among adult males: a randomized controlled trial comparing with acceptance and commitment therapy and treatment as usual
Source: Front Psychiatry. 2023 Aug 22;14:1215963. doi: 10.3389/fpsyt.2023.1215963 (PMC10477784; doi:10.3389/fpsyt.2023.1215963)
Supplement: Supplementary file 4 [file Data_Sheet_4.doc]

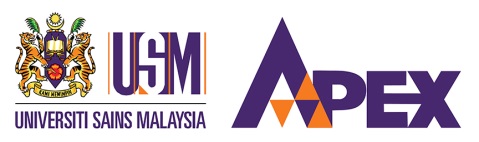


JEPeM-USM

Supplementary appendix 4. Participant information sheet and consent form for this study (English version)

JAWATANKUASA ETIKA PENYELIDIKAN (MANUSIA) – JEPeM USM

UNIVERSITI SAINS MALAYSIA

RESEARCH INFORMATION (TRANSLATED ENGLISH COPY)

Research Title: The Efficacy of Acceptance and Commitment Therapy (ACT) for the Treatment of Alcohol Use Disorder among Adult Males: A Randomized Controlled Trial Comparing with Conventional Therapy and Virtual Reality Exposure Therapy (VRET)

*Name of main and co-Researcher: Dr. Mohammad Farris Iman Leong Bin Abdullah (MMC: 43103, Deng Hongdu*

#### INTRODUCTION

You are invited to take part voluntarily in an interventional research. This research is about observing the severity of alcohol craving through the characteristics of electroencephalogram (EEG; a measuring device connected to the brain to measure the electrical activities of the brain) under various alcohol cue. You are requested to answer several questionnaires such as Alcohol Use Disorders Identification Test (AUDIT), ii) Clinical Institute Withdrawal Assessment-Alcohol, Revised (CIWA-Ar), iii) Visual Analogue Scale (VAS), and iv) Penn Alcohol Craving Scale (PACS), as well as emotional rating scales, such as as i) Hamilton Anxiety Scale (HAMA), and ii) Hamilton Depression Scale (HAMD). You will be treated with conventional treatment for alcohol dependence for the first 2 weeks of admission. In addition, this study will analyze the efficacy of acceptance and commitment therapy (ACT) and vitual reality exposure therapy (VRET), which are two new psychotherapy methods for alcohol addiction, in reducing alcohol craving and preventing relapse.

It is important that you read and understand this research information before agreeing to participate in this study. You will receive a copy of this form to keep for your records if you agree to participate.

Your participation in this study is expected to take about 24 weeks to complete. This study is estimated to include up to 120 participants.

#### PURPOSE OF THE STUDY

The purpose of this study are to determine the EEG characteristics of patients with alcohol dependence while watching alcohol-related visual cues; to determine the relationship between drinking habits, severity of subjective craving and EEG characteristics, and to determine the effectiveness of acceptance and commitment therapy (ACT) and vitual reality exposure therapy (VRET) to alleviate alcohol dependence and preventing relapse. You will be treated with conventional treatment for alcohol dependence for the first 2 weeks of admission and administered several questionnaires such as Alcohol Use Disorders Identification Test (AUDIT), ii) Clinical Institute Withdrawal Assessment-Alcohol, Revised (CIWA-Ar), iii) Visual Analogue Scale (VAS), and iv) Penn Alcohol Craving Scale (PACS), as well as emotional rating scales, such as as i) Hamilton Anxiety Scale (HAMA), and ii) Hamilton Depression Scale (HAMD) to assess craving and emotional state.

#### PARTICIPANTS CRITERIA

The research team members will discussed your eligibility to participate in this study. It is important that you are completely truthful with the staff including your health history.

This study will include individual who are:

• hospitalized patients diagnosed with alcohol use disorder

• male, age 18 to 55 years old, Han nationality, junior high school education or above, right-handed

• those who have not consumed alcoholic beverages in the last 2 weeks

• those with normal eyesight (including corrected vision).

This study will not incude individual who are:

• those with current and lifetime history of abuse of any other psychoactive substances (except tobacco).

• those with current and lifetime history of other mental diseases.

• those with current and lifetime history of central nervous system diseases or serious physical illnesses.

• those who are unable to complete the EEG detection or psychological scale assessment.

• those who are unable to cooperate with ACT or VRET, or who are seriously uncomfortable with psychotherapy.

STUDY PROCEDURES

All participants will be involved in the study in two ways. First is medical treatment related procedures. Initially, you will be given conventional treatment, such as benzodiazepine replacement therapy, low-dose antipsychotics, and adequate vitamin B. This will take 2 weeks

Second, participants will be assessed with a few medical procedures such as EEG monitoring and performed various alcohol-related visual cue tests to detect event-related potentials (ERP). In addition, they will be administered various psychometric assessments to measure alcohol dependence and craving rating scales, such as: i) Alcohol Use Disorders Identification Test (AUDIT), ii) Clinical Institute Withdrawal Assessment-Alcohol, Revised (CIWA-Ar), iii) Visual Analogue Scale (VAS), and iv) Penn Alcohol Craving Scale (PACS), as well as emotional rating scales, such as as i) Hamilton Anxiety Scale (HAMA), and ii) Hamilton Depression Scale (HAMD). Then, blood collection commence to gather blood samples for liver function test, serum electrolytes and γ-glutamyl transpeptidase (GGT) (these assessments are done as baseline assessment). The total duration of the assessment will take 30 minutes.

Third, participants with 2 weeks of abstinence from alcohol. will be randomized into three groups i.e. acceptance and commitment therapy (ACT), vitual reality exposure therapy (VRET), and treatment-as-usual control group. Participants are then re-assessed with ERP, AUDIT, CIWA-Ar, VAS, PACS, HAMA, MAMD, liver function test, serum electrolytes and GGT at 4 weeks (immediately after completion of interventions), 12 weeks, and 24 weeks after baseline assessment.

Below are descriptive of the interventions that will be provided according to the groups you assigned to:

(1) Acceptance and commitment therapy (ACT): It is a third-generation cognitive behavioral approach which uses acceptance and mindfulness processes, and commitment and behavior change processes to produce psychological flexibility. Unlike CBT, which aims to change unhelpful thoughts and feelings, ACT was designed to increase adaptive coping through acceptance, cognitive defusion, mindfulness, and perspective-taking exercises while supporting cancer survivors in aligning behavior with their personal values. Hence, ACT consists of 8 sessions, one session per week, one hour per session to facilitate cancer patients to development and maintenance of health behavioral improvements by targeting internal barriers, such as emotional discomfort and self-defeating thoughts, and by fostering connection and commitment to personal values associated with self-management of positive health behaviors.

(2) Virtual reality exposure therapy (VRET): Each session of VRET will last 25 minutes and consists of three parts: 5 minutes of relaxation, 10 minutes of exposure to high-risk situation and 10 minutes of exposure to aversive situation. The relaxation scene includes four beautiful landscapes, and patients can choose any of the most comfortable landscape. The visual stimulation of high-risk scene is designed as any combination of four different scenes (street barbecue stands, restaurant, bar and home) and four kinds of alcoholic beverages (Chinese liquor, beer, grape wine and cocktail), which are customized according to the patient's personal preference. At the same time, the odor of the alcoholic beverage of the patient's choice will be provided as an olfactory stimulation. The aversive situation will be the visual and auditory stimulation provided by a video of an alcoholic vomiting, while olfactory stimulation will be provided by cotton balls soaked in fermented dairy products. The intervention will be given for 5 sessions per week for a total of 20 sessions. Hence, the total duration of intervention is 4 weeks.

(3) Control group: you will receive non-specific ingredients of the psychotherapeutic approach, such as psychological understanding to the management of an individual patient, identifying current problems, providing opportunities for disclosure, and reassurance. They will be given equal amount of time and attention from the professional figure compared to the intervention groups, whereby they will also attach to a 8-session programme.

RISKS

Participation in this research has minimal risk. Nevertheless, if you experience emotional disturbance after answering the questionnaires, we will recommend reference to counsellor in Advenced Medical and Dental Institute, Universiti Sains Malaysia and 2nd Affiliated Hospital of Xinxiang Medical University, Henan, China. If you still exhibit depressive and anxiety symptoms after you have completed the study, we will recommend reference to Advanced Medical and Dental Institute, Universiti Sains Malaysia and Department of Psychiatry, 2nd Affiliated Hospital of Xinxiang Medical University, Henan, China for further assessment and treatment as necessary. Please inform the research team if you encounter any problems or if you have any important information which will change your participation in the study.

In addition, prior to referral, rescue medication such as benzodiazepine will be administered to calm down patients. In addition, subjects will be provided with counselling services from Henan Mental Hospital, Henan, China if needs arise during the study when they experienced mental disturbances. All subjects will be assured anonymity of personal information when they are offered to participate in the study and assured that all the benefits which they were entitled to will be provided if they decided to withdraw from the study. Finally, the subjects will be recommended for referral to support group, such as Alcoholics Anonymous once they have completed the study.

Specific risk may also occur in different groups of alcohol dependent subjects:

(a) those who are unemployed may also present with financial constrain and difficulty to get a job. We may recommend for referral to social workers for financial aid and for job manager and occupational therapists in the community psychiatry team in Advanced Medical and Dental Institute, Universiti Sains Malaysia and the Department of Psychiatry, 2nd Affiliated Hospital, XXMU for assistance in seeking the right job and for job training.

(b) those who have family members may place the close family members at risk of domestic violence and various psychosocial issues. Hence, we may offer help to family members for mental health screening and referrals to counsellors or Advanced Medical and Dental Institute, Universiti Sains Malaysia and Department of Psychiatry, 2nd Affiliated Hospital, XXMU for further management.

(c) those who are having marital issues with spouse may be recommended for marital counselling under Advanced Medical and Dental Institute, Universiti Sains Malaysia and the Department of Psychiatry, 2nd Affiliated Hospital, XXMU.

(d) those in the placebo group may be at risk of relapse and experience mental disturbances along the course of the study. Rescue medication such as benzodiazepine may be administered to relieve withdrawal symptoms and if you opt to withdraw from the study, immediate referral to Advanced Medical and Dental Institute, Universiti Sains Malaysia and the Department of Psychiatry, 2nd Affiliated Hospital, XXMU for further treatment will be carried out.

REPORTING HEALTH EXPERIENCES.

Please contact, at any time, the following researcher if you experience any health problem either directly or indirectly related to this study.

Dr. Mohammad Farris Iman Leong Bin Abdullah [MMC Registration No._43103___] at +604-5622482 or +6018-6669950.

#### PARTICIPATION IN THE STUDY

Your taking part in this study is entirely voluntary. You may refuse to take part in the study or you may stop your participation in the study at anytime, without any penalty or loss of benefits to which you are otherwise entitled. Your participation also may be stopped by the research team without your consent if in any form you have violated the study eligibility criteria. The research team member will discussed with you if the matter arises.

#### POSSIBLE BENEFITS [Benefit to Individual, Community, University]

The research procedures will be provided to you without any cost. The direct benefit of this study are: (a) You will received information regarding your mental health status which are expected to be important to safeguard your mental health after you were diagnosed with alcohol use disorder. (b) You will also enrolled in psychotherapy sessions in acceptance and commitment therapy (ACT) or virtual reality exposure therapy (VRET) which is expected to help you to maintain your mental health. The study will also help you to maintain abstinence from alcohol and in remission and hence will indirectly improve your daily life, opportunity to gain new permanent job and improve your relationship with your family.

The findings of this study will hopefully bring benefits to the community by providing evidence that the two psychotherapy intervention will alleviate alcohol dependence and prevent relapse. This will allowed recommendation to integrate these two interventions into the treatment regime of alcohol addiction as data on the efficacy of these two interventions among patients with alcohol use disorder is scarce.

You will not receive any compensation from this study. There will be no insurance for participating in this study, but if there is any study-related injury or disability involving the participants and family members, the treatment and rehabilitation expenses will be covered fully by the research team. However you may get reimbursement for your travelling cost while in the study duration. This study does not plan to develop any commercial product from its findings.

#### QUESTIONS

If you have any question about this study or your rights, please contact;

Dr. Mohammad Farris Iman Leong Bin Abdullah

Primary investigator

Department of Community Health

Advanced Medical and Dental Institute

Universiti Sains Malaysia

SAINS@BERTAM

13200 Kepala Batas

Pulau Pinang

Malaysia

+6018-6669950

Deng Hongdu

Co-primary investigator

Department of Community Health

Advanced Medical and Dental Institute

Universiti Sains Malaysia

SAINS@BERTAM

13200 Kepala Batas

Pulau Pinang

Malaysia

(+86) 139 2827 9403

If you have any questions regarding the Ethical Approval or any issue / problem related to this study, please contact;

Mr. Mohd Bazlan Hafidz Mukrim

Secretary of Human Research Ethics Committee USM

Division of Research & Innovation (R&I)

USM Health Campus

Tel. No. : +609-767 2354 / +609-767 2362

Email : [bazlan@usm.my](mailto:bazlan@usm.my)

OR

Miss Nor Amira Khurshid Ahmed

Secretariat of Human Research Ethics Committee USM

Research Creativity & Management Office (RCMO)

USM Main Campus, Penang

Tel. No. : +604-6536537

Email : [noramira@usm.my](mailto:noramira@usm.my)

The investigators serve only as investigator of this study and they are not the one who provide service to the participants.

#### CONFIDENTIALITY

Your information will be kept confidential by the researchers and will not be made publicly available unless disclosure is required by law.

Data obtained from this study that does not identify you individually will be published for knowledge purposes.

Your original records may be reviewed by the researcher, the Ethical Review Board for this study, and regulatory authorities for the purpose of verifying the study procedures and/or data. Your information may be held and processed on a computer. Only research team members are authorized to access your information. The information will be stored for 2 years after completion of the study before it is discarded following standard procedures. While any biological sample (blood and feces) will be discarded following standard procedures once analysis is completed in the study. Any future possible use of the data and specimen collected for research purpose will be communicated to you by the research team and you may refuse to consent for future use and we will discard the data and specimen accrodingly.

The feedback on the study findings will be informed by the research team after completion of the study upon request from the participants.

By signing this consent form, you authorize the record review, information storage and data process described above.

#### SIGNATURES

To be entered into the study, you or a legal representative must sign and data the signature page [ATTACHMENT S or or ATTACHMENT P]

ATTACHMENT S

Subject Information and Consent Form

(Signature Page)

Research Title: The Efficacy of Acceptance and Commitment Therapy (ACT) for the Treatment of Alcohol Use Disorder among Adult Males: A Randomized Controlled Trial Comparing with Conventional Therapy and Virtual Reality Exposure Therapy (VRET)

*Name of main and co-Researcher: Dr. Mohammad Farris Iman Leong Bin Abdullah (MMC: 43103, Deng Hongdu*

To become a part this study, you or your legal representative must sign this page. By signing this page, I am confirming the following:

- I have read all of the information in this Patient Information and Consent Form including any information regarding the risk in this study and I have had time to think about it.
- All of my questions have been answered to my satisfaction.
- I voluntarily agree to be part of this research study, to follow the study procedures, and to provide necessary information to the doctor, nurses, or other staff members, as requested.
- I may freely choose to stop being a part of this study at anytime.
- I have received a copy of this Participant Information and Consent Form to keep for myself.

Participant Name

Participant I.C No

Signature of Participant or Legal Representative Date (dd/MM/yy)

Name of Individual

Conducting Consent Discussion

Signature of Individual Date (dd/MM/yy)

Conducting Consent Discussion

Name & Signature of Witness Date (dd/MM/yy)

Note: i) All participants who are involved in this study will not be covered by insurance.

ATTACHMENT P

Participant’s Material Publication Consent Form

Signature Page

Research Title: The Efficacy of Acceptance and Commitment Therapy (ACT) for the Treatment of Alcohol Use Disorder among Adult Males: A Randomized Controlled Trial Comparing with Conventional Therapy and Virtual Reality Exposure Therapy (VRET)

*Name of main and co-Researcher: Dr. Mohammad Farris Iman Leong Bin Abdullah (MMC: 43103, Deng Hongdu*

To become a part this study, you or your legal representative must sign this page.

By signing this page, I am confirming the following:

- I understood that my name will not appear on the materials published and there have been efforts to make sure that the privacy of my name is kept confidential although the confidentiality is not completely guaranteed due to unexpected circumstances.
- I have read the materials or general description of what the material contains and reviewed all photographs and figures in which I am included that could be published.
- I have been offered the opportunity to read the manuscript and to see all materials in which I am included, but have waived my right to do so.
- All the published materials will be shared among the medical practitioners, scientists and journalist world wide.
- The materials will also be used in local publications, book publications and accessed by many local and international doctors world wide.
- I hereby agree and allow the materials to be used in other publications required by other publishers with these conditions:
- The materials will not be used as advertisement purposes nor as packaging materials.
- The materials will not be used out of contex – i.e.: Sample pictures will not be used in an article which is unrelated subject to the picture.

Participant Name

Participant I.C No. Participant’s Signature Date (dd/MM/yy)

Name and Signature of Individual Date (dd/MM/yy)

Conducting Consent Discussion

Note: i) All participants who are involved in this study will not be covered by insurance.
