# Supplementary material for: The efficacy of virtual reality exposure therapy for the treatment of alcohol use disorder among adult males: a randomized controlled trial comparing with acceptance and commitment therapy and treatment as usual
Source: Front Psychiatry. 2023 Aug 22;14:1215963. doi: 10.3389/fpsyt.2023.1215963 (PMC10477784; doi:10.3389/fpsyt.2023.1215963)
Supplement: Supplementary file 5 [file Data_Sheet_5.doc]

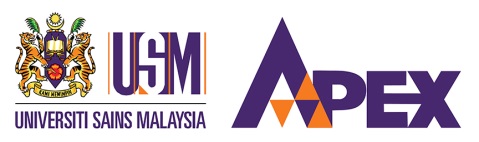


JEPeM-USM

Supplementary appendix 5. Participant information sheet and consent form for this study (Chinese version)

研究伦理委员会（人类） – JEPeM USM

马来西亚理科大学

研究信息（中文译本）

研究标题： 接纳承诺疗法（ACT）治疗成年男性酒依赖患者的疗效：一项与传统疗法和虚拟现实暴露疗法（VRET）比较的随机对照试验

*主要研究人员及合作研究人员**：穆罕默德·法里斯·伊曼·梁·阿卜杜拉 博士（MMC: 43103），邓红都*

#### 介绍

我们邀请您自愿参与本项干预性研究。本项目是关于通过脑电图（EEG；一种连接到大脑以测量脑区电活动的测量工具）的特征来观察酒精渴求严重程度的研究，主要是检测处于各种酒精线索下的脑电图。同时，您需要回答几份问卷，例如：酒精使用障碍筛查量表（AUDIT）、临床酒精戒断评定量表（CIWA-AR）、视觉模拟标尺（VAS）和宾夕法尼亚酒精渴求量表（PACS）；以及情绪评定量表例如：汉密尔顿焦虑量表（HAMA）和汉密尔顿抑郁量表（HAMD）。在入院前两周，您将接受酒依赖的常规治疗。此外，本研究将分析接纳承诺疗法（ACT）和虚拟现实暴露疗法（VRET）这两种新的酒精成瘾心理治疗方法在减少酒精渴求以及预防复发方面的疗效。

在同意参加本研究之前，您必须阅读并理解本研究信息。如果您同意参加，您将收到一份本表格的副本以备记录。

您参与的本研究预计需要约24周时间完成。本研究预计入组共计120名受试者。

#### 研究目的

本研究旨在测定酒依赖患者在观察酒精相关视觉线索时的脑电特征；确定酒依赖患者饮酒习惯、主观渴求严重程度与脑电特征之间的关系；以及评估接纳承诺疗法（ACT）和虚拟现实暴露疗法（VRET）在缓解酒精依赖和预防复发方面的疗效。在入院前两周，您将接受酒依赖的常规治疗，并进行一些问卷调查用于评估渴求和情绪状态，例如：酒精使用障碍筛查量表（AUDIT）、临床酒精戒断评定量表（CIWA-AR）、视觉模拟标尺（VAS）和宾夕法尼亚酒精渴求量表（PACS）；以及情绪评定量表例如：汉密尔顿焦虑量表（HAMA）和汉密尔顿抑郁量表（HAMD）。

#### 受试者标准

研究团队成员将讨论您参与本研究的资格。请您务必对工作人员完全诚实，包括您的健康史。

本研究将纳入符合以下标准的受试者：

• 被诊断为酒精使用障碍的住院患者；

• 男性，年龄18至55岁，汉族，初中及以上学历，右利手；

• 在过去2周内未饮用酒精性饮料；

• 视力正常（包括矫正视力）。

本研究将排除符合以下标准的受试者：

• 有其他精神活性物质的滥用史（烟草除外）；

• 有其他精神疾病病史；

• 有中枢神经系统疾病或严重躯体疾病病史；

• 不能够配合完成脑电检测或心理量表评估；

• 不能够配合接受ACT或VRET干预，或者对心理治疗严重不适者。

研究流程

所有入组受试者的研究开展主要包含两方面。第一是药物治疗相关过程。首先，受试者将接受常规住院治疗，主要包括苯二氮卓类药物替代疗法、低剂量抗精神病药物和足量的维生素B。这将需要两周时间。

然后，受试者将通过一些医学检测工具进行评估，例如脑电图监测，并进行各种酒精相关视觉线索测试以检测事件相关电位（ERP）。同时，他们还将接受各种心理测量以衡量酒精依赖和渴求程度，例如：酒精使用障碍筛查量表（AUDIT）、临床酒精戒断评定量表（CIWA-AR）、视觉模拟标尺（VAS）和宾夕法尼亚酒精渴求量表（PACS）；以及情绪评定量表例如：汉密尔顿焦虑量表（HAMA）和汉密尔顿抑郁量表（HAMD）。然后，收集血液样本用于检测肝功能、血清电解质和γ-谷氨酰转肽酶（GGT）（上述这些评估均作为基线评估）。评估的总时间约为30分钟。

最后，受试者经过为期两周的酒精脱瘾治疗。他们将被随机分为三组，即接纳承诺疗法（ACT）组、虚拟现实暴露疗法（VRET）组和常规治疗对照组。在基线评估后的第4周（干预结束后立即）、12周和24周对受试者再次进行ERP、AUDIT、CIWA-Ar、VAS、PACS、HAMA、HAMD、肝功能、血清电解质和GGT的检测评估。

以下是本研究的干预性措施（根据您被分到的研究小组所提供）的描述性说明：

（1）接纳承诺疗法（ACT）：它是第三代的认知行为疗法，通过接纳和正念过程、承诺和行为改变过程来产生心理灵活性。与CBT不同，ACT旨在改变无益的想法和感受，通过接纳、认知解离、正念和换位思考练习来增加适应性应对，同时支持酒依赖患者将行为与他们的个人价值观调整统一。因此，ACT包括8次课程，每周一次，每次一小时，通过针对内部障碍促进酒依赖患者健康行为改善的发展与维持，并通过培养积极健康行为自我管理建立与个人价值相关的联系和承诺。

（2）虚拟现实暴露疗法（VRET）：每次的虚拟现实暴露疗法干预持续25分钟，包括三个部分：5分钟的放松、10分钟的高风险情境暴露和10分钟的厌恶情境暴露。放松场景为四处优美景观，受试者可选择任一感觉最舒适的景观。高风险场景的视觉刺激是四个不同的场景（街头烧烤摊、餐馆、酒吧、家中）和四种酒精性饮料（中国白酒、啤酒、葡萄酒、鸡尾酒）的任意组合，根据受试者个人喜好定制，同时给予患者所选择的酒精性饮料的气味提供嗅觉刺激。厌恶情境是由一系列酒精性呕吐的视频提供的视觉及听觉刺激，以及由由浸泡在发酵乳制品中的棉球提供提供的嗅觉刺激。每周进行5次干预，共计20次。因此，干预的总持续时间为4周。

（3）对照组：你将接受心理治疗方法的非特定成分，如对患者个体管理的心理理解，确定当前的问题，提供披露的机会，以及安慰。与干预组相比，他们将从专业人士那里得到同等的时间和关注，因此他们也将参加一个8次课程的方案。

风险

参与本项研究的风险很小。但是如果您在回答问卷后出现情绪障碍，我们建议您向中国河南新乡医学院第二附属医院的咨询师进行咨询。如果您在研究结束后仍出现抑郁和焦虑症状，我们建议您到新乡医学院第二附属医院的精神科接受进一步评估及治疗。如果您遇到任何问题或获得任何会改变您参与研究的重要信息，请告知本研究团队。

此外，在转介前还会给患者继续服用苯二氮卓类抢救性药物，以使患者平静下来。此外，如果受试者在研究过程中遇到精神障碍，将会提供来自中国河南省精神病医院的咨询服务。当所有受试者被邀请参与研究时，他们将被保证不透露个人信息，并保证如果他们决定退出研究，他们将获得他们有权获得的所有福利。最后，一旦受试者完成研究，他们将被推荐转介到支持小组，如匿名戒酒协会。

具体的风险也可能发生在不同的酒依赖群体中：

（a）失业人士可能会存在经济上的限制，难以找到工作。我们可能建议转介给社会工作者以获得经济援助，并建议转介给新乡医学院第二附属医院精神科社区精防团队的工作经理和职业治疗师，以帮助他们寻找合适的工作和进行职业培训。

（b）有家庭成员的人士可能会使其亲密家庭成员面临家庭暴力及各种心理社会问题的风险。因此，我们可以帮助家属进行心理健康检查，并转介到心理咨询师或新乡医学院第二附属医院精神科进一步管理。

（c）与配偶有婚姻问题的人士，我们可能建议他们到新乡医学院第二附属医院精神科接受婚姻咨询。

（d）对照组的患者可能有复发的风险，并在研究过程中出现精神障碍。可以使用苯二氮卓类药物来缓解酒精戒断症状，如果您选择退出研究，将立即转介到新乡医学院第二附属医院精神科进一步治疗。

健康问题报告

如果您遇到任何与本研究直接或间接相关的健康问题，请随时联系以下研究人员：

穆罕默德·法里斯·伊曼·梁·阿卜杜拉（博士）[MMC编号 43103 ] 电话：+604-5622482 / +6018-6669950。

#### 参与研究

您的参与是完全自愿的。您可以在任何时候拒绝参加本研究或停止参加本次研究，您本应享有的利益不会受到任何处罚或损失。如果您以任何形式违反了研究资格标准，研究团队也可能会在未经您同意的情况下停止您的参与。如果出现问题，研究团队成员将与您讨论。

#### 潜在获益 [个人、社会、大学的受益]

整个研究程序将免费提供给您。本研究的直接好处包括：（a）在您被诊断为酒精使用障碍后，您将获得有关您的精神健康状况的信息，这些信息对维护您的精神健康非常重要；（b）您还将参加接纳承诺疗法（ACT）或虚拟现实暴露疗法（VRET）等心理治疗课程，这将帮助您保持心理健康。本项研究还将帮助您维持长期戒酒，从而间接改善您的日常生活，有机会获得新的固定工作和改善您与家人的关系。

本研究的发现有望通过提供证据证明两种心理治疗干预将减轻酒精依赖和预防复发，从而带来社会效益。这将允许建议将这两种干预措施整合到酒精成瘾的治疗方案中，而目前这两种干预措施在酒精使用障碍患者中的疗效数据是稀缺的。

您不会从本次研究中获得任何报酬。参加本次研究不提供保险，但如果受试者及其家属在研究过程中发生任何与研究相关的伤害或残疾，治疗和康复费用将由研究团队全额承担。但是，您可以在研究期间获得车旅费报销。本研究不打算根据其发现开发任何商业产品。

#### 答疑

如果您对本研究或您的权利有任何疑问，请联系我们：

穆罕默德·法里斯·伊曼·梁·阿卜杜拉 博士

主要研究人员

公共卫生系

高级医学和牙科研究所

马来西亚理科大学

SAINS@BERTAM

13200 加巴拉巴打

槟城

马来西亚

+6018-6669950

邓红都

共同主要研究人员

公共卫生系

高级医学和牙科研究所

马来西亚理科大学

SAINS@BERTAM

13200 加巴拉巴打

槟城

马来西亚

(+86) 139 2827 9403

如果您对伦理审批或与本研究相关的任何问题有任何疑问，请联系：

穆罕默德·巴兹兰·哈菲兹·穆克里姆 先生

马来西亚理科大学人类研究伦理委员会 秘书

研究与创新部（R&I）

马来西亚理科大学健康学院

电话号码：+609-767 2354 / +609-767 2362

邮箱：[bazlan@usm.my](mailto:bazlan@usm.my)

或者

诺尔·阿米拉·胡尔希德·艾哈迈德 女士

马来西亚理科大学人类研究伦理委员会秘书处

研究创新与管理办公室（RCMO）

马来西亚理科大学主校区，槟城

电话号码：+604-6536537

邮箱：[noramira@usm.my](mailto:noramira@usm.my)

研究人员只是本项目的研究开展者，并不是为受试者提供服务的人。

#### 保密性

研究人员将对您的信息保密，除非法律要求披露，否则不会公开。

从本研究中获得的数据不会披露您的个人身份，数据将出于知识目的发表。

您的原始记录可能会被研究人员、本研究的伦理审查委员会和监管机构审查，以核实研究程序和/或数据。您的信息可能会在计算机上储存和处理。只有研究团队成员有权查阅您的信息。这些信息将在研究完成后存储两年，然后按照标准程序弃置。而任何生物样本(血液和粪便)将在研究分析完成后按照标准程序弃置。本研究团队将会告知您相关资料及样本出于研究目的的日后使用情况，您可以拒绝日后使用，我们亦会相应弃置该资料及样本。

在研究结束后，应受试者的要求，研究团队将告知研究结果的反馈。

签署本知情同意书，即表示您已授权上述的记录审查、信息存储和数据处理。

#### 签字

同意参与本研究，您或法定代理人必须在签名页上签字并填写资料。

[附件 S 或 附件 P]

附件 S

受试者信息及知情同意书

（签字页）

研究标题： 接纳承诺疗法（ACT）治疗成年男性酒依赖患者的疗效：一项与传统疗法和虚拟现实暴露疗法（VRET）比较的随机对照试验

*主要研究人员及合作研究人员：穆罕默德·法里斯·伊曼·梁·阿卜杜拉 博士（MMC: 43103），邓红都*

加入本研究前，您或您的法定代理人须在本页签名。通过签署本页，本人确认以下内容：

- 我已经阅读了这份受试者信息及知情同意书中的全部内容，包括关于本研究风险的所有信息，并且我已在足够的时间内对以上内容进行思考。
- 我对于本研究相关的所有问题都得到了满意回答。
- 我自愿同意参与本研究，遵循研究程序，并根据要求向医生、护士或其他工作人员提供必要的信息。
- 我可以随时自由选择停止参与这项研究。
- 我已收到受试者信息及知情同意书的副本，并由我本人保存。

受试者姓名

受试者编号

受试者（或法定代理人）签字 日期（年/月/日）

个人姓名

进行同意讨论

个人签字 日期（年/月/日）

进行同意讨论

见证人姓名及签字 日期（年/月/日）

备注： i) 所有参与本研究的受试者将不在保险范围内。

附件 P

受试者资料发表同意书

签字页

研究标题： 接纳承诺疗法（ACT）治疗成年男性酒依赖患者的疗效：一项与传统疗法和虚拟现实暴露疗法（VRET）比较的随机对照试验

*主要研究人员及合作研究人员：穆罕默德·法里斯·伊曼·梁·阿卜杜拉 博士（MMC: 43103），邓红都*

加入本研究前，您或您的法定代理人须在本页签名。通过签署本页，本人确认以下内容：

- 我已知晓本人的姓名将不会出现在出版的资料上。同时，即使由于不可预估情况而无法完全保障保密性，研究人员已经努力确保本人姓名的隐私保密。
- 我已经阅读了资料或资料所包含的一般描述，并查阅了所有包括我在内的可能被发表的照片和图像。
- 我已经被提供机会阅读论文手稿，并查看所有包含我的资料，但我放弃了这样做的权利。
- 本研究所有出版的资料将在全世界的医疗从业者、科学家和记者之间共享。
- 本研究的资料也将被用于国内外医学杂志出版物及图书出版物，并且供全世界范围内的医生查阅。
- 我同意并允许在符合以下条件的其他出版商的出版物中使用本研究的资料：
- 研究资料的内容将不会被用于广告目的或产品包装。
- 不得断章取义地使用研究资料——例如：样本图片不得被用于与图片无关的文章中。

受试者姓名

受试者编号 受试者签字 日期（年/月/日）

个人姓名及签字 日期（年/月/日）

进行同意讨论

备注： i) 所有参与本研究的受试者将不在保险范围内。
